# Reply to Comment on: “Based on a Single Case, Cardiopulmonary Training Cannot Be Recommended as Therapy for Hemiparesis in MELAS With Ischemic Stroke”

**DOI:** 10.1002/kjm2.70295

**Published:** 2026-09-24

**Authors:** Yen‐Sen Lu, Ko‐Long Lin

**Affiliations:** ^1^ Department of Physical Medicine and Rehabilitation Kaohsiung Medical University Hospital, Kaohsiung Medical University Kaohsiung Taiwan; ^2^ Department of Physical Medicine and Rehabilitation, Faculty of Medicine, College of Medicine Kaohsiung Medical University Kaohsiung Taiwan

Dear Editor,

1

We appreciate the thoughtful comments on our recent case report entitled “Cardiopulmonary Exercise Testing (CPET) Guided Sub‐Anaerobic Threshold Rehabilitation in MELAS Syndrome: A Case Report” [1]. These comments provide an opportunity to clarify several important clinical and neuroimaging findings.

Regarding the distinction between an ischemic stroke (IS) and a stroke‐like episode (SLE), our diagnosis of IS was supported by neuroimaging findings. His brain magnetic resonance imaging (MRI) demonstrated an “acute infarction in the ventral portion of the right medulla,” and the subsequent computed tomography angiography (CTA) confirmed the “occlusion of right vertebral artery (VA) V4 after posterior inferior cerebellar artery (PICA) level.” Because the infarction corresponded to a defined vascular territory, it is distinct from the typical non‐vascular distribution of SLEs. Furthermore, the MRI revealed “diffuse arteriosclerotic change of intracranial arteries,” while the CTA revealed no evidence of arterial dissection, aneurysm, or other structural vascular abnormality. Taken together, these imaging findings suggest that the stroke was more likely attributable to conventional atherosclerosis associated with the patient's type 2 diabetes and hyperlipidemia, rather than primary mitochondrial vasculopathy.

We fully agree that a single case report cannot definitively establish causality or be universally generalized. While spontaneous neurological recovery likely contributed to his functional gains, the specific physiological adaptations observed cannot be fully explained by natural recovery alone. Following the 12‐week intervention, the patient demonstrated an increased workload at anaerobic threshold (AT) and a progressive decline in resting serum lactate, with an unchanged peak oxygen uptake. These findings are consistent with previous studies demonstrating improved mitochondrial efficiency following aerobic training in patients with mitochondrial myopathy [2, 3].

Finally, we acknowledge the absence of specific parameters like the heteroplasmy rate, which reflects the limitations inherent to routine clinical practice. Our intention is not to advocate CPET as a standard therapy, but rather to highlight its value as a personalized evaluation tool. By defining a safe metabolic window, CPET may help prevent systemic metabolic stress during rehabilitation in patients with impaired aerobic oxidative metabolism. We hope these clarifications provide additional context for our clinical rationale and stimulate further prospective studies evaluating the broader role of CPET in this patient population.

Once again, we extend our sincere gratitude to the authors for their keen interest in our publication and for taking the time to share their valuable perspectives. Such constructive academic dialogues are essential for advancing the clinical care of complex diseases.

## Conflicts of Interest

All authors take responsibility for all aspects of the reliability and freedom from bias of the data presented and their discussed interpretation.

## Data Availability

The data that support the findings of this study are available from the corresponding author upon reasonable request.
